# Eligibility of real-life patients with COPD for inclusion in trials of inhaled long-acting bronchodilator therapy

**DOI:** 10.1186/s12931-016-0433-5

**Published:** 2016-09-23

**Authors:** David M. G. Halpin, Marjan Kerkhof, Joan B. Soriano, Helga Mikkelsen, David B. Price

**Affiliations:** 1 Department of Respiratory Medicine, Royal Devon & Exeter Hospital, Exeter, EX2 5DW UK; 2 Research in Real-Life Ltd, 5a Coles Lane, Oakington, Cambridge, CB24 3BA UK; 3 Instituto de Investigación Hospital Universitario de la Princesa (IISP), Universidad Autónoma de Madrid, Cátedra UAM-Linde, Madrid, Spain; 4 Cambridge Research Support Ltd, Warren House, Aylsham, NR11 5UN UK; 5 Academic Primary Care, Division of Applied Health Sciences, University of Aberdeen, Polwarth Building, Foresterhill, Aberdeen, AB25 2ZD UK

**Keywords:** Randomised controlled trial, Real-life research, Chronic obstructive pulmonary disease, Long-acting bronchodilator

## Abstract

**Background:**

Management guidelines of chronic obstructive pulmonary disease (COPD) are mainly based on results of randomised controlled trials (RCTs), but some authors have suggested limited representativeness of patients included in these trials. No previous studies have applied the full range of selection criteria to a broad COPD patient population in a real-life setting.

**Methods:**

We identified all RCTs of inhaled long-acting bronchodilator therapy, during 1999–2013, at ClinicalTrials.gov and translated trial selection criteria into definitions compatible with electronic medical records. Eligibility was calculated for each RCT by applying these criteria to a uniquely representative, well-characterised population of patients with COPD from the Optimum Patient Care Research Database (OPCRD).

**Results:**

Median eligibility of 36 893 patients with COPD for participation in 31 RCTs was 23 % (interquartile range 12–38). Two studies of olodaterol showed the highest eligibility of 55 and 58 %. Conversely, the lowest eligibility was observed in two studies that required a history of exacerbations in the past year (3.5 and 3.9 %). For the patient subgroup with modified Medical Research Council score ≥2, the overall median eligibility was 27 %.

**Conclusions:**

By applying an extensive range of RCT selection criteria to a large, representative COPD patient population, this study highlights that the interpretation of results from RCTs must take into account that RCT participants are variably, but generally more representative of patients in the community than previously believed.

**Electronic supplementary material:**

The online version of this article (doi:10.1186/s12931-016-0433-5) contains supplementary material, which is available to authorized users.

## Background

Chronic obstructive pulmonary disease (COPD) is a debilitating disorder that has become a major public health concern worldwide [1–3]. Guidelines for COPD management and treatment are predominantly based on results from double-blind, placebo-controlled, randomised controlled trials (RCTs). Generally considered to be the optimal study design to test the efficacy and safety of medical interventions [2], RCTs are designed to answer specific questions about treatments. This requires a uniform and well-characterised patient population, supervised care, careful monitoring, and control of factors that may confound or modify any potential effects [4]. However, this stringent selection means that RCT findings may be limited in the extent to which treatment effects can be extrapolated to a broad general patient population [4, 5]. For example, real-life patients with COPD tend to be older than trial participants, either because RCTs restrict the age range, or because they exclude patients with comorbidities [6], and the latter are sometimes excluded despite drug effectiveness being influenced by comorbidities [7].

Treatment of most patients with COPD occurs under very different conditions from RCTs, where a multitude of factors may influence the real-life effectiveness of therapeutic options [8]. This gap between community patients and RCTs leads to the so-called clinician’s fallacy, in which an inaccurate view of the nature and causes of a disease results from studying a small number of cases in clinical trials [9]. The process of care in clinical trials may also influence the assessment of treatment efficacy: for example, guidelines for COPD management recommend that patients with COPD are seen 1–2 times a year [10], while many RCTs improve adherence through much more frequent visits [11, 12]. Real-life adherence to treatment is not only low, but also influenced by side effects, which may in turn influence patients’ response to medication [13–15].

Long-acting bronchodilators are currently one of the first choices of maintenance medication for COPD according to guidelines. These can be used alone, together with other bronchodilators, or in combination with inhaled corticosteroids (ICS) [2, 10]. However, little is known about how representative participants of the RCTs testing bronchodilators are of the general COPD patient population. Despite this, there is a widespread and frequently quoted assumption that over 90 % of people treated for COPD would be ineligible to participate in RCTs [16–18]. To our knowledge, five studies have addressed this question and have reported eligibility ranges from 5 to 42 % [16, 19–22]. These studies were all limited, either by considering a low number of patients [16, 19, 21], by selecting patient populations that are likely not representative of community patients with COPD [16, 21, 22], or by only considering a limited range of selection criteria employed by RCTs [16, 19–21]. Individual studies show that the full range of criteria relevantly affect eligibility for participation in RCTs. There is therefore a need to combine the evidence provided by previous studies in order to give the full picture of eligibility, as well as to study any potential changes in trends [23].

A comprehensive assessment of the relevance of study findings to general patient populations requires thorough description of patient selection and clinical management [5]. The aim of this study was to determine the proportion of the general UK patient population with COPD that would be eligible for inclusion in recent RCTs testing inhaled long-acting bronchodilators. The objectives were; 1) to give an overview of inclusion and exclusion criteria applied in relevant clinical trials, 2) to describe the frequency of the characteristics and conditions of these selection criteria in a general COPD patient population, identified from a large database of anonymised medical records, and 3) to determine the percentage of patients with COPD in the database who would meet the eligibility criteria for RCTs of inhaled long-acting bronchodilator therapy.

## Methods

### Selection of RCTs and study population

We selected RCTs investigating the effects of inhaled long-acting bronchodilators in COPD from studies registered at https://clinicaltrials.gov/ through to 13 October 2014, using the criteria listed in Table 1. Briefly, eligible RCTs were phase III or phase IV trials in which spirometry parameters, COPD exacerbations, or COPD mortality were the primary efficacy outcome. For further details on search terms, see the online supplement.

**Table 1:** Criteria employed for selection of RCTs from ClinicalTrials.gov, and patients from OPCRD

Selection criteria for RCTs
a. Recruiting patients with COPD
b. Phase III or Phase IV randomised, double-blind, placebo-controlled trial, testing i. Long-acting muscarinic antagonist (LAMA) OR ii. Long-acting β-agonist (LABA) OR iii. LAMA/LABA combination
c. Primary outcomes were i. spirometry parameter(s) OR ii. COPD exacerbations OR iii. mortality
d. Duration of treatment was ≥24 weeks post-randomisation
e. Enrolled ≥100 patients with COPD
f. Medicine was tested at licensed dose
Selection of patients with COPD from the OPCRD database
a. Quality and Outcomes Framework (QOF) approved diagnostic code of COPD, which includes the requirement for a post-bronchodilator FEV_1_/FVC <0.70 [31]
b. Registered in OPCRD with data extracted from general practice at least once after 1 January 2011. The index date was defined as the date of the last data extraction
c. ≥1 year of data available prior to the index date to define RCT inclusion/exclusion criteria
d. FEV_1_ and FVC recorded within 5 years of the index date
e. mMRC score recorded within 5 years of index date
f. Recorded valid blood eosinophil count ever
g. Age ≥40 years

*Abbreviations*: *COPD* chronic obstructive pulmonary disease, *FEV*_*1*_ forced expiratory volume in 1 s, *FVC* forced vital capacity, *mMRC score* modified Medical Research Council score [2]

The population used to test the effects of selection criteria was patients in the Optimum Patient Care Research Database (OPCRD) [24], aged ≥40 years, with a confirmed diagnosis of COPD, as well as data on forced expiratory volume in 1 s (FEV_1_), modified Medical Research Council (mMRC) score [2] and full blood counts (Table 1). The index date was defined as the date of the last data extraction from the general practice.

### Data source

The OPCRD is a quality-controlled, longitudinal, respiratory-focused database that contains de-identified data from general practices across the UK [24]. The database contains information about patient management in primary and secondary care and combines electronic patient records with linked patient-reported data, which are collected using disease-specific questionnaires. Routine clinical data are extracted from general practice management systems and include patient demographic characteristics, comorbidities, exacerbation history, mMRC score and current therapy. At the time of the study, the OPCRD contained 44,376 patients with a diagnostic Read code for COPD recorded who had data extracted from practice at least once from January 2011 to January 2015. The database has been approved by the Trent Multicentre Research Ethics Committee for clinical research use (approval reference 10/H0405/3). The study was approved by the Anonymised Data Ethics Protocols and Transparency committee, which is the independent scientific advisory committee for the OPCRD, and the study protocol was registered with the European Network of Centres for Pharmacoepidemiology and Pharmacovigilance (registration number ENCEPP/SDPP/7727).

### Methods of analysis

To identify and analyse the data, a stepwise method was employed (see Additional file 1). Briefly, all inclusion and exclusion criteria reported in eligible RCTs were collected, and all published manuscripts and protocols were checked for additional selection criteria. Eligibility criteria were then divided into categories, which were translated into definitions of criteria compatible with the OPCRD database (Additional file 2: Tables S1 − S7). These criteria were applied to the database patient population with complete data on FEV_1_, full blood counts, and mMRC score, and the results were reported as mean (range) and/or median (interquartile range [IQR]). The percentage of OPCRD patients eligible for inclusion in each RCT was calculated for two reference populations: the full eligible population of patients with COPD, and a subpopulation with mMRC score ≥2, ie patients who have symptoms of moderate dyspnea and comprise a more specific target population for treatment with long-acting bronchodilators. Eligibility time-trends were studied by dividing the RCTs by start year using three 5-year periods, and differences were assessed using Kruskal-Wallis Test.

## Results

### Inclusion/exclusion criteria reported in RCTs

**Fig. 1:**
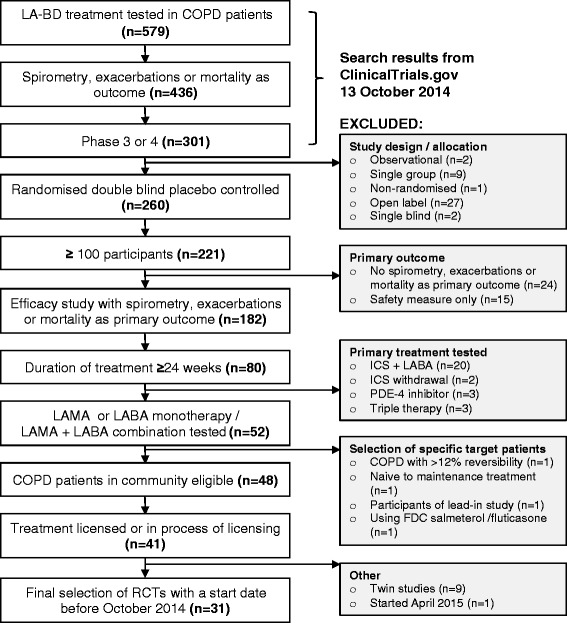
Selection of clinical trials. Flow chart showing the criteria and stepwise selection process of eligible randomised controlled trials (RCTs). Search parameters are outlined in the supplementary methods. Abbreviations: COPD = chronic obstructive pulmonary disease; FDC = fixed-dose combination; ICS = inhaled corticosteroids; LA-BD = long-acting bronchodilator; LABA = long-acting β-agonist; LAMA = long-acting muscarinic antagonist; PDE-4 = phosphodiesterase-4; RCT = randomised controlled trial

Using the selection process outlined in Fig. 1, 31 RCTs were studied (Table 2). These trials, which had start dates between February 1999 and July 2013, tested three long-acting muscarinic antagonists (LAMA; tiotropium, aclidinium and glycopyrronium), three long-acting β-agonists (LABA; formoterol, indacaterol and olodaterol), and three LABA/LAMA combinations (indacaterol + glycopyrronium, vilanterol + umeclidinium and tiotropium + olodaterol). Eighteen trials (58 %) were carried out in the last 5 years of the study period (2009–2013). FEV_1_ was the primary outcome in 29 (94 %) of the trials, while the remaining two [25, 26] studied reduction of exacerbation rates.

**Table 2:** Selected RCTs, sorted by experimental drug tested, start date and publication date

Generic name drug tested	RCT	Trial name	Sample size	Twin study^a^	Start date^c^	Publication date	% Male	Age mean ± SD or median (IQR)	FEV_1_ % predicted mean ± SD	Reference
Tiotropium	NCT02172287		410	NCT02173691	Feb-99	Jul-02	75	64.9 ± 7.9	Not available	[32]
	NCT00274014		1010		Oct-00	Mar-06	88	64.8 ± 9.3	35.6 ± 12.6	[27]
	NCT00274547		1829		Sep-01	Sep-05	99	67.9 ± 8.6	39.4 ± 13.5	[33]
	NCT00277264		913		Jan-02	Nov-07	60	66.9 ± 8.9	39.4 ± 12.0	[34]
	NCT00144339	UPLIFT	5993		Dec-02	Oct-08	75	64.5 ± 8.5	45.3 ± 13.6	[12]
	NCT00387088		3991	NCT00168844/NCT00168831	Sep-06	Oct-10	78	64.8 ± 9.1	49.3 ± 13.2	[35]
	NCT00563381	POET-COPD	7376		Jan-08	Mar-11	75	62.4 ± 9.0	48.3 ± 13.9	[36]
	NCT01126437		17 135		May-10	Oct-13	72	65.0 ± 9.1	47.9 ± 12.7	[37]
	NCT01455129^b^		839		Nov-11	Feb-14	Not available			[38]
Formoterol	NCT00134979		847		Oct-04	Nov-08	78	62.6 ± 8.9	51.2 ± 13.1	[39]
Aclidinium	NCT00363896	ACCLAIM/COPD I	843	NCT00358436	Aug-06	Apr-11	79	62.3 ± 8.3	53.6 ± 15.2	[40]
	NCT01001494	ATTAIN	828		Oct-09	Oct-12	67	62.4 ± 8.0	52.5 ± 14.1	[41]
	NCT01044459		605		Nov-09	Dec-13	58	63.6 ± 9.7	52.3 ± 13.2	[42]
Indacaterol	NCT00463567		1683		Apr-07	Jul-10	63	63.6 ± 9.1	55.6 ± 14.7	[43]
	NCT00567996		1002		Nov-07	Jun-11	75	63.6 ± 8.8	53.3 ± 13.9	[44]
	NCT00792805		563		Nov-08	Feb-14	94	65.4 ± 8.8	49.9 ± 12.1	[45]
	NCT00845728	INVIGORATE	3444		Mar-09	Sep-13	77	64.0 (40–91)	40.5 ± 6.0	[46]
Olodaterol	NCT00782210		624	NCT00782509	Nov-08	Jun-14	73	64.9 ± 8.4	48.9 ± 15.4	[28]
	NCT00793624		904	NCT00796653	Jan-09	Jul-14	78	63.8 ± 8.7	51.2 ± 14.7	[29]
Glycopyrronium	NCT00929110	GLOW2	1066		Jun-09	Nov-12	64	63.7 ± 8.8	56.0 ± 13.3	[47]
	NCT01005901	GLOW1	822		Oct-09	Dec-11	82	63.9 ± 9.2	54.5 ± 12.9	[48]
	NCT01566604	GLOW7	460		Mar-12	Jan-15	96	64.8 ± 8.1	51.0 ± 12.4	[49]
Indacaterol + Glycopyrronium	NCT01120691	SPARK	741		Apr-10	May-13	75	63.3 ± 8.0	37.2 ± 8.1	[25]
	NCT01202188	SHINE	2144		Sep-10	Dec-13	75	64.0 ± 8.8	55.2 ± 13.1	[50]
	NCT01315249	ILLUMINATE	259		Mar-11	Mar-13	71	63.3 ± 8.0	60.3 ± 10.6	[51]
	NCT01709903	LANTERN	676		Nov-12	June-15	91	65.1 ± 7.9	51.8 ± 12.9	[52]
	NCT01782326	LANTERN			Jul-13	Recruiting				[26]
Vilanterol + Umeclidinium	NCT01313650		1532	NCT01313637	Mar-11	Oct-13	71	63.0 ± 8.9	47.4 ± 13.1	[53]
	NCT01316900		1141	NCT01316913	Mar-11	Jun-14	69	62.9 ± 9.0	47.7 ± 13.0	[54]
	NCT01777334		905		Jan-13	Dec-14	68	62.3 ± 8.5	46.4 ± 12.9	[55]
Tiotropium + Olodaterol	NCT01431274		2624	NCT01431287	Sep-11	Apr-15	73	64.0 ± 8.3	45.0 ± 11.7	[30]

^a^A twin study is a RCT registered at ClinicalTrial.gov with identical design, selection criteria and primary outcomes of efficacy. ^b^This trial was the only one to apply an upper age limit, 85 years. ^c^Start date registered on ClinicalTrials.gov

An overview of RCT eligibility criteria is shown in the supplement (Additional file 3: Table S8; Additional file 4: Table S9; Additional file 5: Table S10). Briefly, all trials included patients aged ≥40 years with a smoking history of ≥10 pack years. Patients with mild airflow limitation (%predicted FEV_1_ ≥ 80), which was found in 19 % of OPCRD patients, were excluded from all trials. Patients with severe airflow limitation (%predicted FEV_1_ < 30), found in 4 % of patients, were excluded from half of them, and most trials excluded patients with a recent history of exacerbations (*n* = 25). Other frequently applied COPD-related exclusion criteria included oxygen treatment (*n* = 23), recent participation in a pulmonary rehabilitation program (*n* = 16) and use of maintenance oral corticosteroids (*n* = 12). Finally, all trials excluded patients with asthma, all but one [27] excluded patients with concomitant pulmonary disease, and all excluded patients with other clinically significant diseases using diverse methodology.

### Distribution of reported criteria in the OPCRD population

**Fig. 2:**
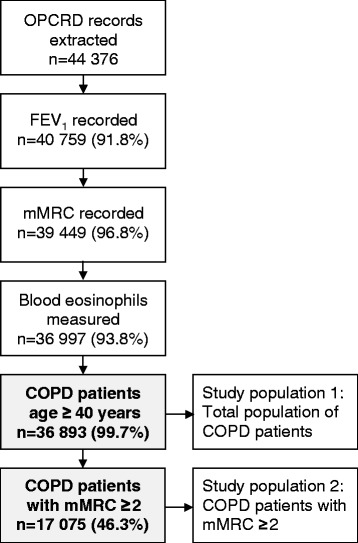
Selection of study population. Patient flow chart showing selection of study population from the Optimum Patient Care Research Database (OPCRD). Abbreviations: COPD = chronic obstructive pulmonary disease; FEV_1_ = forced expiratory volume in one second; mMRC = modified Medical Research Council score [2]

Using the selection criteria listed in Table 1, 36 893 eligible patients were identified in the OPCRD database (Fig. 2). Demographic and clinical characteristics of this population are shown in Table 3, and the distribution of other applied criteria is shown in the supplement (Additional file 6: Tables S11 − S17).

**Table 3:** Characteristics of OPCRD patients with COPD that fulfil study criteria in Table 1 (*n* = 36 893)

Characteristic	
Age, mean (SD)	71.3 (10.6)
Male, n (%)	19 478 (52.8)
FEV_1_ % predicted, mean (SD)	62.5 (20.0)
GOLD category of airflow limitation, n (%)	
GOLD 1: FEV_1_ ≥ 80 %	19.3 % (7 118)
GOLD 2: 50 % ≤ FEV_1_ < 80 %	53.5 % (19 755)
GOLD 3: 30 % ≤ FEV_1_ < 50 %	23.0 % (8 468)
GOLD 4: FEV_1_ < 30 %	4.2 % (1 552)
mMRC score grade ≥2, n (%)	17 075 (46.3)
Pack years of smoking, n (%):	
≥ 10	34 758 (94.2)
≥ 20	34 232 (92.8)
Maintenance therapy^a^ prescribed within 6 months, n (%)	25 594 (69.4)
Symptomatic^b^, n (%)	29 579 (80.2)
History of ≥ 1 exacerbation in the last year, n (%)	18 373 (49.8)

*Abbreviations*: *COPD* chronic obstructive pulmonary disease, *FEV*_*1*_ forced expiratory volume in 1 s, *GOLD* Global Initiative for Chronic Obstructive Lung Disease [2], *ICS* inhaled corticosteroids, *LABA* long-acting β-agonists, *LAMA* long-acting muscarinic antagonists, *LTRA* leukotriene receptor antagonists, *mMRC score* modified Medical Research Council score [2]

^a^ICS, LAMA, LABA, LTRA or phosphodiesterase inhibitors

^b^mMRC grade ≥2, or maintenance therapy prescribed within 6 months

Substantial differences were identified between RCT participants and patients with COPD in the OPCRD database. OPCRD patients were on average 7 years older than RCT participants (71 and 64 years, respectively), and they were less frequently male (53 % vs 76 %). The %predicted mean FEV_1_ was also substantially lower for RCT participants than for OPCRD patients (49 and 63 %, respectively).

Of the selected OPCRD patients with COPD, almost half had mMRC score ≥2 [2], 69 % had recorded prescriptions of maintenance therapy and 50 % had a history of COPD exacerbation within the past year.

### Proportion of OPCRD patients eligible for inclusion in RCTs

The overall median eligibility of OPCRD patients with COPD to participate in RCTs was 23 % (IQR 12–38), mean 24 % (range 3.5–58 %, Tables 4, 5 and 6). Studies of olodaterol [28, 29] showed the highest eligibility (55 and 58 %), while studies of combination therapies showed the lowest overall eligibility (13 %, Table 6), which was primarily due to all but one [30] being restricted to patients with evidence of COPD symptoms. As expected, the two studies of indacaterol + glycopyrronium that focused on reducing exacerbations in a patient population with a history of exacerbations in the past year [25, 26], showed the lowest inclusion of 3.5 and 3.9 %, respectively.

**Table 4:** Percentage^a^ of total OPCRD patients with COPD eligible for RCTs testing tiotropium (*n* = 36 893)

Step	Criterion for sequential selection	NCT02172287	NCT00274014	NCT00274547	NCT00277264	NCT00144339	NCT00387088	NCT00563381	NCT01126437	NCT01455129	Median
1	FEV_1_	46.8	50.3	46.8	56.3	65.8	46.8	65.8	65.8	72.8	56.3
2	Other inclusion criteria	38.5	20.6	44.5	23.2	54.4	44.5	26.1	55.6	64.7	44.5
3	COPD-related exclusion criteria	37.8	19.9	41.6	21.8	53.4	43.8	25.8	54.4	59.8	41.6
4	Concomitant pulmonary disease	35.6	19.9	41.6	20.2	47.3	41.1	25.5	48.2	52.9	41.1
5	Asthma, allergic diseases and atopy	23.8	14.3	32.9	14.7	37.5	29.8	19.6	38.2	39.1	29.8
6	Comorbidities	16.7	11.2	30.7	9.6	28.8	27.9	15.2	29.3	31.4	27.9
7	Other relevant conditions	15.7	11.2	30.7	9.5	27.1	27.9	14.4	27.5	29.2	27.1
8	Final eligible proportion (%)	15.7	11.2	25.1	9.5	22.5	27.8	11.8	22.9	25.0	22.5

*Abbreviations*: *COPD* chronic obstructive pulmonary disease, *FEV*_*1*_ forced expiratory volume in 1 s

^a^Stepwise reduction of the percentage of the total number of OPCRD patients with COPD eligible for individual RCTs (columns) when applying groups of criteria (rows) sequentially. Median values for all RCTs shown in the table are listed in the last column

**Table 5:** Percentage^a^ of OPCRD patients with COPD eligible for RCTs testing other single treatments^b^ (*n* = 36 893)

Step	Criterion for sequential selection	NCT00134979 (F^a^)	NCT00363896 (A^a^)	NCT01001494 (A)	NCT01044459 (A)	NCT00463567 (I^a^)	NCT00567996 (I)	NCT00792805 (I)	NCT00845728 (I)	NCT00782210 (O^a^)	NCT00793624 (O)	NCT00929110 (G^a^)	NCT01005901 (G)	NCT01566604 (G)	Median
1	FEV_1_	63.9	80.7	76.5	76.5	76.5	76.5	76.5	23.0	80.7	80.7	76.5	76.5	76.5	76.5
2	Other inclusion criteria	51.4	68.3	64.9	64.9	71.4	72.3	72.3	12.5	76.4	76.4	59.1	72.3	57.8	68.3
3	COPD related exclusion criteria	50.5	67.1	64.3	63.8	57.8	68.8	65.0	10.1	75.6	74.9	56.5	70.2	51.1	64.3
4	Concomitant pulmonary disease	44.8	60.8	58.3	57.9	52.6	62.4	58.3	8.8	74.8	70.9	51.0	62.7	46.2	58.3
5	Asthma, allergic diseases and atopy	34.7	43.8	46.0	45.7	42.0	44.6	46.0	6.8	72.5	66.5	38.0	49.2	34.2	44.6
6	Comorbidities	26.5	42.6	44.7	38.2	32.0	36.0	43.3	6.5	55.0	61.9	28.3	47.8	25.7	38.2
7	Other relevant conditions	26.3	41.5	44.7	38.2	31.7	34.9	43.3	6.5	55.0	57.6	28.1	47.8	25.2	38.2
8	Final eligible proportion (%)	26.3	41.5	44.6	38.1	26.2	34.9	43.3	6.5	55.0	57.6	23.1	39.2	20.7	38.1

*Abbreviations*: *COPD* chronic obstructive pulmonary disease, *FEV*_*1*_ forced expiratory volume in 1 s

^a^Stepwise reduction of the percentage of the total number of OPCRD patients with COPD eligible for individual RCTs (columns) when applying groups of criteria (rows) sequentially. Median values for all RCTs shown in the table are listed in the last column. ^b^Other single treatments include: formoterol (F), aclidinium (A), indacaterol (I), olodaterol (O) or glycopyrronium (G)

**Table 6:** Percentage^a^ of total OPCRD patients with COPD eligible for RCTs testing combined treatments^b^ (*n* = 36 893)

Step	Criterion for sequential selection	NCT01120691 (I + G^a^)	NCT01202188 (I + G)	NCT01315249 (I + G)	NCT01709903 (I + G)	NCT01782326 (I + G)	NCT01313650 (V + U^a^)	NCT01316913 (V + U)	NCT01777334 (V + U)	NCT01431287 (T + O^a^)	Median
1	FEV_1_	27.2	76.5	67.6	76.5	43.5	65.8	65.8	65.8	80.7	65.8
2	Other inclusion criteria	15.0	59.1	50.2	33.9	13.8	32.9	32.9	32.9	76.4	32.9
3	COPD related exclusion criteria	9.5	42.7	18.4	21.5	10.7	28.5	28.2	31.8	74.9	28.2
4	Concomitant pulmonary disease	8.4	38.2	16.7	19.4	9.4	25.3	25.1	27.8	70.9	25.1
5	Asthma, allergic diseases and atopy	5.4	24.9	11.4	14.9	7.0	20.0	24.4	27.0	68.7	20.0
6	Comorbidities	4.1	19.2	9.0	11.2	5.0	14.8	18.2	20.1	52.2	14.8
7	Other relevant conditions	4.0	18.5	8.9	11.1	4.8	13.7	16.7	18.5	48.9	13.7
8	Final eligible proportion (%) eligible	3.5	15.2	7.2	8.6	3.9	13.7	13.2	14.7	48.9	13.2

*Abbreviations*: *COPD* chronic obstructive pulmonary disease, *FEV*_*1*_ forced expiratory volume in 1 s

^a^Stepwise reduction of the percentage of the total number of OPCRD patients with COPD eligible for individual RCTs (columns) when applying groups of criteria (rows) sequentially. Median values for all RCTs shown in the table are listed in the last column

^b^Combined treatments include: indacaterol and glycopyrronium (I + G), vilanterol and umeclidinium (V + U), and tiotropium and olodaterol (T + O)

**Fig. 3:**
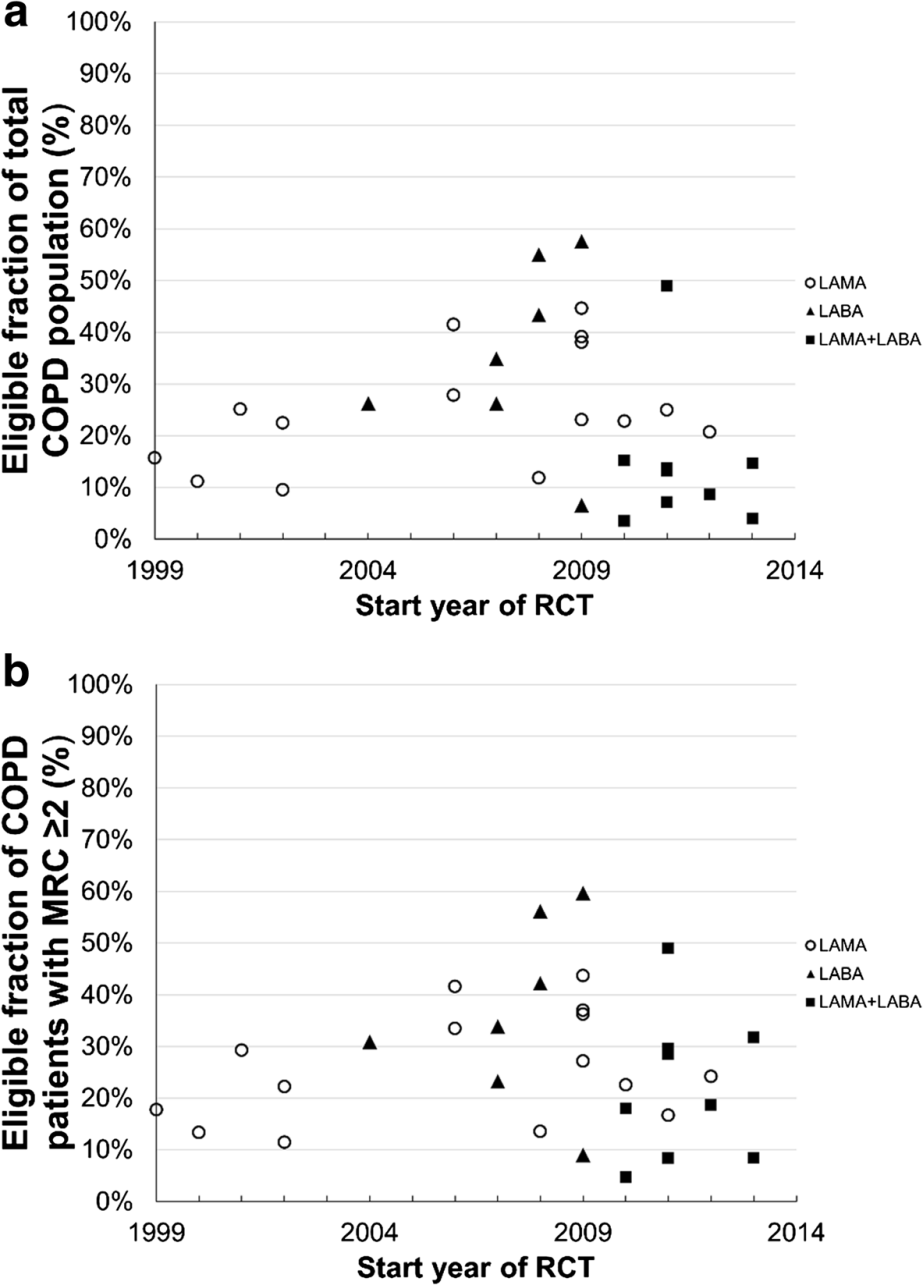
Eligibility to participate in randomised controlled trials (RCT). Eligibility of patients from the Optimum Patient Care Research Database (OPCRD) to be included in individual randomised controlled trials (RCTs) by RCT start year. Data show fraction of; **a** total population of patients with chronic obstructive pulmonary disease (COPD) and **b** patients with COPD and modified Medical Research Council (mMRC) score ≥2. Data on trials investigating long-acting β-agonists (LABA) are indicated by ○, long-acting muscarinic antagonists (LAMA) by ▲, and LABA + LAMA combination therapy by ■

The overall median eligibility (IQR) in the first 5-year period (1999–2003) was 16 % (10–24 %) when five RCTs testing tiotropium were carried out. This rose to 31 % (26–43 %) in the second 5-year period (2004–2008), when trials of other single therapies were carried out, and decreased to 18 % (8–38 %) in the final 5-year period (Fig. 3a). No significant difference was observed in overall eligibility across the 15-year period (*p* = 0.076). The relatively high mean eligibility in 2004–2008 could mainly be attributed to more relaxed selection criteria for the level of FEV_1_ in studies testing single therapies other than tiotropium (Tables 5 and 6).

A subanalysis of OPCRD patients with more severe dyspnea (mMRC score ≥2) [2] showed that median eligibility increased from 23 to 27 % (range 4.7–60 %) compared with the overall population (Additional file 6: Tables S18–S20). A small average increase in eligibility of 1.0 and 0.6 % was observed for RCTs testing tiotropium or other single therapies respectively, while the eligibility for RCTs testing combination therapies increased by an average of 7.5 % compared with the overall population (Fig. 3b).

## Discussion

Using the selection criteria reported by 31 RCTs and applying them to a broad UK primary care population, this study showed that the overall median eligibility of patients with COPD to participate in RCTs of inhaled long-acting bronchodilators was 23 % (IQR 12–38). The highest eligibility was identified in two studies of olodaterol (55 and 58 %). Conversely, the lowest eligibility was identified in two trials of indacaterol + glycopyrronium that required a history of frequent exacerbations (3.5 and 3.9 %). Some variation was observed in eligibility over time (1999–2013) with a mean eligibility of 16, 31 and 18 % in the first, second and third 5-year periods respectively, although no significant difference was observed over the whole 15-year period. A subanalysis of patients with more severe dyspnea (mMRC score ≥2), who would likely be the patients treated in practice, showed an overall median eligibility of 27 %.

Five studies have previously investigated the eligibility of patients with COPD for participation in RCTs of bronchodilators [16, 19–22]. In agreement with our results, these studies reported that the majority of community patients with COPD would be excluded from RCTs, although the proportion varied substantially. Our data agree with the findings by Kruis et al [20] that RCT participants are on average younger, more likely to be male, and have lower %predicted FEV_1_ compared with the COPD patient population. Although previous studies have highlighted important limitations of the generalisability of COPD-related RCTs, they themselves suffer from substantial limitations that raise questions about the generalisabilty of their findings. The first is the representativeness of the chosen patient population. Four previous studies considered a low number of patients (110–696) [16, 19, 21, 22], which likely limits the accuracy of their estimates. Of these, two [21, 22] considered patients from hospital clinics, who likely have more severe disease on average than patients seen in primary care. A third study [16] identified patients with COPD in a postal survey of randomly selected adults in the community. However, the response rate was low (21 %), and many of those identified as having COPD had not previously been diagnosed, which was likely one of the main reasons for the low reported eligibility. The second important limitation of previously published studies is the range of eligibility criteria considered. Four of them [16, 19–21] considered a limited range of eligibility criteria. Of these, one [21] only considered criteria from a single RCT and another [19] did not specify how the criteria were chosen. Finally, only two previous studies [16, 20] provided information on eligibility for participation in individual RCTs.

Compared with the above, the current study has several strengths. In the UK all patients are registered with a General Practitioner (GP) who holds records that include demographic information, disease and comorbidity diagnoses as Read codes, prescribing information, test results, and information related to secondary care visits and hospitalisations. The OPCRD database is a large UK community database containing anonymised research-quality data focused on respiratory disease, derived from these GP records. Firstly, the database enabled us to assess the eligibility of patients for RCTs in a large and uniquely representative UK patient population with a diagnosis of COPD that meets the requirements of the Quality and Outcomes Framework (QOF), which is the UK system for the performance management and payment of general practitioners [31]. The results are therefore more likely to be truly representative of community patients with COPD than previously published studies. Secondly, we carried out an unbiased and comprehensive search among all trials registered at ClinicalTrials.gov and identified 579 studies testing long-acting bronchodilators in patients with COPD. From these, we selected 31 double-blind, placebo-controlled RCTs according to pre-specified criteria, extracted all reported selection criteria and translated them into definitions compatible with electronic medical records. This resulted in a range of selection criteria that was as close to the original RCTs as practically possible, which therefore likely provides better estimates of true eligibility. An example of this is that, while the study by Kruis et al [20] considered a large and likely representative COPD patient population (*n* = 3 508), the study only applied common inclusion criteria based on spirometry, smoking status and previous COPD exacerbations, but did not consider exclusion criteria such as COPD-related characteristics, the presence of asthma, atopy or other clinically relevant diseases. In the case of the UPLIFT trial [12], this resulted in a dramatic difference in eligibility, which was found to be 23 % in our analysis and 42 % in that by Kruis et al. [20] Conversely, Travers et al. [16] employed similar criteria to our study, but they also reported potential use of medication as one of the most common exclusion criteria. The current study did not exclude people on this basis and, in order to study the representativeness of the full COPD patient population, assumed that all patients would be capable of undergoing a washout.

Although information for this study was collected from both ClinicalTrials.gov and published literature, it is limited by the fact that not all criteria could be completely translated into definitions that match routine point-of-care data, and by potentially incomplete reporting of criteria in trial protocols. In addition to explicit selection criteria, clinicians recruiting patients for RCTs may use their own covert criteria to exclude patients who are more difficult to manage, are housebound, or have multiple comorbidities. Furthermore, some real-life data (eg spirometry) may be less accurately recorded in primary care than during RCTs. We selected patient records with a diagnostic Read code for COPD recorded after April 2008, when a post-bronchodilator FEV_1_/FVC <0.70 was introduced as part of the QOF diagnostic procedure for UK general practitioners and therefore available in all patients [31]. We also selected records with complete data on the applied criteria, and patients may therefore not be fully representative of all patients with COPD registered in the database. For example, eosinophil counts were missing in 7.9 % of extracted records (6.2 % of records with data on lung function and mMRC score). Another potential limitation is that exclusion criteria that refer to recent events (eg exacerbations or respiratory infections in the past 6 weeks) may be a temporary reason for exclusion that does not permanently exclude patients from RCT participation. To address this, we carried out a sensitivity analysis that included patients that would otherwise have been excluded by COPD-related criteria in the last 3 months, and found that this only increased eligibility by 1 %. Finally, this study focused on the representativeness of the patient population, but did not study other aspects that cause RCTs to differ from the real-life ecology of care [5].

Despite these potential limitations, we believe the current study provides the most comprehensive picture to date of the eligibility of real-life patients for participation in RCTs of inhaled long-acting bronchodilators. Our results show that, overall, around a quarter of community patients with COPD are eligible for RCT participation. Some studies represent less than 4 % of patients, leading to a high risk of “clinician’s fallacy”, while the most representative studies include over half of the real-life patient community.

## Conclusions

This study combines an extensive range of RCT selection criteria with a large, representative COPD patient population to provide detailed information on eligibility of patients with COPD for participation in RCTs. The results highlight that interpretation of outcomes from RCTs of inhaled long-acting bronchodilators therapy in COPD must take into account that RCT participants are variably representative of real-life patients. In order to assess the relevance of the results of RCTs, it is essential that there is full and accurate reporting of trial selection criteria in published manuscripts and in clinical trial databases. This analysis also emphasises that, in addition to the results of RCTs, complementary information from effectiveness studies of real-life patients with COPD should be an important consideration for future guideline development.

## Additional files

Additional file 1: Supplementary methods. (DOCX 19 kb) Additional file 2: Tables S1-S7. Applied definitions of inclusion criteria in the Optimum Patient Care Research Database (OPCRD). **Table S2.** Applied definitions of COPD-related exclusion criteria in the Optimum Patient Care Research Database (OPCRD). **Table S3.** Applied definitions of exclusion criteria related to concomitant pulmonary disease in the Optimum Patient Care Research Database (OPCRD). **Table S4.** Applied definitions of exclusion criteria related to asthma, allergic disease and atopy in the Optimum Patient Care Research Database (OPCRD). **Table S5.** Applied definitions of exclusion criteria related to clinically significant diseases other than COPD, asthma or allergic disease, in the Optimum Patient Care Research Database (OPCRD). **Table S6.** Applied definitions of exclusion criteria related to other relevant conditions in the Optimum Patient Care Research Database (OPCRD). **Table S7.** Applied definitions of exclusion criteria for specific contra-indications in the Optimum Patient Care Research Database (OPCRD). (DOCX 17 kb) Additional file 3: Table S8. Overview of inclusion and exclusion criteria of RCTs testing tiotropium. (XLSX 16 kb) Additional file 4: Table S9. Overview of inclusion and exclusion criteria of RCTs testing for RCTs testing other single treatments. (XLSX 17 kb) Additional file 5: Table S10. Overview of inclusion and exclusion criteria of RCTs testing combined treatments. (XLSX 16 kb) Additional file 6: Tables S11-S20. Distribution of inclusion criteria in the Optimum Patient Care Research Database (OPCRD) population. **Table S12.** Distribution of COPD-related exclusion criteria in the population of patients with COPD in the Optimum Patient Care Research Database (OPCRD). **Table S13.** Distribution of concomitant pulmonary diseases in the population of patients with COPD in the Optimum Patient Care Research Database (OPCRD). **Table S14.** Distribution of asthma, allergic diseases and atopy in the population of patients with COPD in the Optimum Patient Care Research Database (OPCRD). **Table S15.** Distribution of other comorbidities in the population of patients with COPD in the Optimum Patient Care Research Database (OPCRD). **Table S16.** Distribution of other relevant conditions in the population of patients with COPD in the Optimum Patient Care Research Database (OPCRD). **Table S17.** Distribution of contra-indications in the population of patients with COPD in the Optimum Patient Care Research Database (OPCRD). **Table S18.** Percentage of Optimum Patient Care Research Database (OPCRD) patients with COPD and mMRC ≥2 who would be eligible for RCTs testing tiotropium (*n* = 17 075). **Table S19.** Percentage of Optimum Patient Care Research Database (OPCRD) patients with COPD and mMRC ≥2 (*n* = 17 075) who would be eligible for RCTs testing formoterol (F), aclidinium (A), indacaterol (I), olodaterol (O) and glycopyrronium (G). **Table 20.** Percentage of Optimum Patient Care Research Database (OPCRD) patients with COPD and mMRC ≥2 (*n* = 17 075) who would be eligible for RCTs testing indacaterol + glycopyrronium (I + G), vilanterol + umeclidinium (V + U) and tiotropium + olodaterol (T + O). (DOCX 27 kb)

## Abbreviations

COPD: Chronic obstructive pulmonary disease
FEV_1_: Forced expiratory volume in 1 s
GOLD: Global initiative for chronic obstructive lung disease
GP: General practitioner
ICS: Inhaled corticosteroids
IQR: Interquartile range
LABA: Long-acting beta-agonist
LAMA: Long-acting muscarinic antagonist
LTRA: Leukotriene receptor antagonist
mMRC score: Modified Medical Research Council score
OPCRD: Optimum Patient Care Research Database
QOF: Quality and Outcomes Framework
RCT: Randomised controlled trial
SD: Standard deviation
