# Supplementary material for: Eligibility of real-life patients with COPD for inclusion in trials of inhaled long-acting bronchodilator therapy
Source: Respir Res. 2016 Sep 23;17:120. doi: 10.1186/s12931-016-0433-5 (PMC5034631; doi:10.1186/s12931-016-0433-5)
Supplement: Additional file 1: — Supplementary methods. (DOCX 19 kb) [file 12931_2016_433_MOESM1_ESM.docx]

# Supplementary methods

## Search procedure to select randomised controlled trials (RCT)

The following search terms were used to identify RCT on https://clinicaltrials.gov/

(COPD OR Chronic Obstructive Pulmonary Disease) [CONDITION]

AND

("Long acting muscarinic antagonist" OR LAMA OR "Long acting B2 agonist" OR LABA OR salmeterol OR formoterol OR olodaterol OR vilanterol OR indacaterol OR tiotropium OR aclidinium OR glycopyrronium OR NVA237 OR QAB149 OR "BI 1744" OR BI1744 OR umeclidinium OR GSK573719 OR GW642444 OR QVA149) [TREATMENT]

AND

("lung function" OR spirometry OR FEV OR "Forced Expiratory Volume" OR FVC OR "Forced Vital Capacity" OR PEF OR "peak expiratory flow“ OR exacerbation or mortality) [OUTCOME]

AND

("Phase 3" OR "Phase 4") [PHASE]

## Analysis methods

To identify and analyse relevant data, the following steps were employed:

1. All inclusion / exclusion criteria reported at ClinicalTrials.gov were collected, and manuscripts and protocols published in international journals were checked for additional selection criteria. Results from all studies were published in manuscripts, except one study that was not yet finished (NCT01782326, Table 1).
2. Inclusion and exclusion criteria that had been applied in RCTs were grouped into the following main categories:
   1. Inclusion of patients based on range of FEV_1_
   2. Selection on other inclusion criteria than FEV_1_
   3. Exclusion of patients with COPD-related characteristics
   4. Exclusion of patients with concomitant pulmonary disease
   5. Exclusion of patients with asthma, allergic diseases, atopy or eosinophilia
   6. Exclusion of patients with other comorbidities
   7. Exclusion on other relevant conditions
   8. Exclusion of patients with contra-indications of LAMA and/or LABA
3. Inclusion and exclusion criteria reported in RCTs were translated into OPCRD definitions of criteria. The definitions of criteria applied in the OPCRD database can be found in supplementary Tables S1-7.
4. The distribution of the criteria was calculated in the total OPCRD population with complete data on FEV_1_, blood eosinophil counts and mMRC score.
5. The step by step reduction of the percentage of OPCRD patients eligible for each selected RCT were calculated for the groups of criteria mentioned above for two reference populations:
   1. Total population of OPCRD patients with COPD
   2. Subpopulation of OPCRD patients with mMRC score grade 2 or higher, who have symptoms of dyspnea and are therefore a more specific target population for treatment with long-acting bronchodilators
6. Time-trends in eligibility were studied by dividing the RCTs by start year using three periods of 5 years

# References

1. Disease GIfCOL. Global strategy for the diagnosis, management and prevention of chronic obstructive pulmonary disease. 2016. p. 112.

2. (HSCIC) HSCIC. Quality and Outomes Framework (QOF). http://www.hscic.gov.uk/qof (accessed 08/10/2015 2015).
