# Supplementary material for: Eligibility of real-life patients with COPD for inclusion in trials of inhaled long-acting bronchodilator therapy
Source: Respir Res. 2016 Sep 23;17:120. doi: 10.1186/s12931-016-0433-5 (PMC5034631; doi:10.1186/s12931-016-0433-5)
Supplement: Additional file 2: Tables S1-S7. — Applied definitions of inclusion criteria in the Optimum Patient Care Research Database (OPCRD). Table S2. Applied definitions of COPD-related exclusion criteria in the Optimum Patient Care Research Database (OPCRD). Table S3. Applied definitions of exclusion criteria related to concomitant pulmonary disease in the Optimum Patient Care Research Database (OPCRD). Table S4. Applied definitions of exclusion criteria related to asthma, allergic disease and atopy in the Optimum Patient Care Research Database (OPCRD). Table S5. Applied definitions of exclusion criteria related to clinically significant diseases other than COPD, asthma or allergic disease, in the Optimum Patient Care Research Database (OPCRD). Table S6. Applied definitions of exclusion criteria related to other relevant conditions in the Optimum Patient Care Research Database (OPCRD). Table S7. Applied definitions of exclusion criteria for specific contra-indications in the Optimum Patient Care Research Database (OPCRD). (DOCX 17 kb) [file 12931_2016_433_MOESM2_ESM.docx]

# Supplementary tables

**Table S1** Applied definitions of inclusion criteria in the Optimum Patient Care Research Database (OPCRD)

| **RCT inclusion criterion** | **Definition applied in OPCRD patients with COPD** |
| --- | --- |
| Range of FEV_1_ % predicted | Range of FEV_1_% predicted recorded at latest spirometry within 5 years prior to index date |
| Pack years of smoking  ≥10 / ≥ 20 | Read code for pack years indicates >10 or registered as current or ex-smoker after age 30 / 40 years |
| mMRC ≥2 | Last recorded mMRC ≥2 |
| Symptomatic in diary | Last recorded mMRC ≥2 or maintenance therapy prescribed within 6 months |
| Relatively stable COPD | No COPD exacerbations in the last 6 weeks and no changes in therapeutic regimen for COPD in the last 2 months |
| COPD exacerbation last year | ≥1 COPD exacerbation 12 months prior to index date |
| Able to perform spirometry | No spirometry exclusion Read code after latest spirometry |
| Able to inhale medication | Not applied |
| Able to record in diary | No illiteracy Read code recorded ever |

Abbreviations: COPD = chronic obstructive pulmonary disease; FEV_1_ = forced expiratory volume in 1 second; mMRC = modified Medical Research Council score.{Disease, 2016 #374}

**Table S2** Applied definitions of COPD-related exclusion criteria in the Optimum Patient Care Research Database (OPCRD)

| **RCT exclusion criterion** | **Definition in OPCRD** |
| --- | --- |
| COPD exacerbation within 4 / 6 weeks | ≥1 COPD exacerbations within 4 / 6 weeks prior to index date |
| Respiratory infection within 4 / 6 weeks | ≥1 GP consultations for URTI or LRTI (any Read code) within 4 / 6 weeks prior to index date |
| Upper respiratory infection within 6 weeks | ≥1 GP consultations for URTI (any Read code) within 6 weeks prior to index date |
| Hospitalisation for COPD exacerbations or pneumonia within 3 months / 1 year | ≥1 lower-respiratory hospitalisation within 3 months / 1 year prior to index date |
| Antibiotics for respiratory tract infections (without time range) | ≥1 antibiotics prescription with GP consultation for URTI or LRTI (any Read code) within 6 weeks prior to index date |
| Long term use of antibiotics | More than 9 prescriptions for antibiotics within 12 months to index date |
| Oral steroids use at unstable doses last 6 weeks or at stable doses of ≥10 / ≥20 mg/day) | >6 courses of high dose (≥10 / ≥20 mg/day) oral steroids in year prior to index date |
| Depot corticosteroids last 12 months | Any Read code for prescription of depot corticosteroids therapy within 12 months prior to index date |
| Oxygen treatment for (>1 / >12 / >15 hrs/day, "regular use", "long-term use") | Any Read code for treatment with oxygen therapy within 12 months prior to index date |
| Active in a pulmonary rehabilitation program within 4 weeks / 6 weeks / 1 year | Any Read code for pulmonary rehabilitation within 4 weeks / 6 weeks / 1 year |
| Regular use of short-acting bronchodilators (SA-BD) or patients unable to withhold SA-BDs at least 4 hours | Not applied |
| Change of maintenance treatment within 6 weeks | No new respiratory therapy in the last 6 weeks (ICS, LABA, LAMA, LTRA or PDE-4 inhibitors) |
| Current use of oral sympaticomimetics | Any Read code for oral sympaticomimetics prescription in last 3 months prior to index date |
| Previous treatment with the investigational drugs | Not applied |
| History of non-compliance to drugs | ICS adherence: MPR <70% in last 12 months for patients with first ICS prescription >12 months prior to the index date; otherwise not applied |

Abbreviations: COPD = chronic obstructive pulmonary disease; GP = general practice; ICS = inhaled corticosteroids; LABA = long-acting β-agonist; LAMA = long-acting muscarinic antagonist; LRTI = lower respiratory tract infection; LTRA = leukotriene receptor antagonist; MPR = medication possession ratio; PDE-4 = phosphodiesterase-4 inhibitor; RCT = randomised controlled trial; URTI = upper respiratory tract infection.

**Table S3** Applied definitions of exclusion criteria related to concomitant pulmonary disease in the Optimum Patient Care Research Database (OPCRD)

| **RCT exclusion criterion** | **Definition in OPCRD** |
| --- | --- |
| Concomitant pulmonary disease | Diagnosis Read code for cystic fibrosis, bronchiectasis, active tuberculosis, pulmonary fibrosis, lung disease due to external agents or unspecified lung diseases |
| Clinically evident bronchiectasis | Diagnosis Read code bronchiectasis ever |
| Lung lobectomy or transplantation | Read code for lung lobectomy or transplant ever |
| Active tuberculosis | Diagnosis Read code for tuberculosis in the last 12 months |
| Cystic fibrosis | Diagnosis Read code for cystic fibrosis ever |
| Lung cancer ever | Diagnosis Read code for lung cancer ever |
| Life-threatening pulmonary obstruction ever | Read code for asphyxia / severe asthma attack ever |
| Pulmonary embolism ever | Diagnosis Read code for pulmonary embolism ever |
| α_1_-antitrypsine deficiency | Diagnosis Read code for α_1_-antitrypsine deficiency ever |

**Table S4** Applied definitions of exclusion criteria related to asthma, allergic disease and atopy in the Optimum Patient Care Research Database (OPCRD)

| **RCT exclusion criterion** | **Definition in OPCRD** |
| --- | --- |
| History of asthma | QOF diagnosis Read code for asthma ever and no code for asthma being resolved after that |
| Current asthma | QOF diagnosis Read code for asthma within 12 months prior to index date and no code for asthma being resolved after that |
| Asthma symptoms onset <40 years | Not applied |
| Blood eosinophilia (>400/μl or >600/μl) | Blood eosinophil count >400/μl or >600/μl |
| Allergic rhinitis | Read code for allergic rhinitis within 5 years prior to index date |
| Allergic rhinitis treated with H1 antagonist or intra-nasal corticosteroids intermittently | Read code for allergic rhinitis and prescription for H1 antagonist or intranasal corticosteroids within 5 years prior to index date |
| Atopic eczema | Read code for atopic eczema within 5 years prior to index date |
| Use of antihistamines | Read code for antihistamines within 12 months prior to index date |
| Cromolyn sodium or nedocromil sodium use | Read code for cromolyn sodium or nedocromil sodium prescription within 12 months prior to index date |
| Atopy | Read code for allergic asthma, allergic rhinitis, positive allergy test ever |

Abbreviations: QOF = quality and outcomes framework{(HSCIC), #344}

**Table S5** Applied definitions of exclusion criteria related to clinically significant diseases other than COPD, asthma or allergic disease, in the Optimum Patient Care Research Database (OPCRD)

| **RCT exclusion criterion** | **Definition in OPCRD** |
| --- | --- |
| Clinically significant disease other than COPD that would put the patient at risk or a disease that would influence the results or patients ability to participate | Fulfilling any of the exclusion criteria of diseases mentioned below, except malignancy within 5 years |
| Unstable cardiac conditions | Read codes for myocardial infarction, angina pectoris, unstable cardiac arrhythmia or heart failure within 12 months prior to index date |
| Unstable angina pectoris | Read codes for unstable angina pectoris within 12 months prior to index date |
| Life-threatening (or unstable) cardiac arrhythmia in past year (not excluding stable atrial fibrillation) | Read code for life-threatening or unstable cardiac arrhythmia within 12 months prior to index date or any code ever with a change in drug treatment within 12 months |
| Paroxysmal atrial fibrillation | Read code for paroxysmal atrial fibrillation within 12 months prior to index date |
| Paroxysmal tachycardia (>100) | Read code for paroxysmal tachycardia within 12 months prior to index date |
| Myocardial infarction in past 6 /12 months | Read code for myocardial infarction within 6 / 12 months prior to index date |
| Hospitalisation for heart failure (NYHA class III or IV) | Generic hospitalisation Read code recorded on the same day as Read code for heart failure within 12 months |
| Left ventricular failure (NYHA class III or IV) | Read code for left ventricular failure within last 12 months |
| Current use of beta-blockers | Read code for prescription of beta-blockers in the last 3 months |
| Malignancy treated with resection, radiation or chemotherapy (except local skin carcinoma) within 5 years | Read code for any malignancy within 5 years prior to index date, except Read code for skin carcinoma |
| Diabetes mellitus type 1 or uncontrolled type 2 | Read code for type 1 diabetes mellitus (DM); OR Read code for type 2 DM plus Read code for medication prescription plus Read code indicating no control within 12 months prior to index date |
| Untreated thyrotoxicosis | Read code for thyrotoxicosis within 3 months prior to index date |
| Untreated hypothyroidism | Not applied |
| Hyperadrenergic state | Read code for pheochromocytoma within 12 months prior to index date |
| Mental illness | Read code for depression, anxiety disorder, schizophrenia, lithium therapy or dementia within 12 months prior to index date |
| Tricyclic antidepressants or Monoamine oxidase inhibitors use | Prescription of tricyclic antidepressants or monoamine oxidase inhibitors within 12 months prior to index date |
| Uncontrolled infection due to HIV and/or active hepatitis | Read code for HIV or hepatitis within 12 months prior to index date |

Abbreviations: COPD = chronic obstructive pulmonary disease; HIV = human immunodeficiency virus; NYHA = New York Heart Association.

**Table S6** Applied definitions of exclusion criteria related to other relevant conditions in the Optimum Patient Care Research Database (OPCRD)

| **RCT exclusion criterion** | **Definition in OPCRD** |
| --- | --- |
| Pregnancy, lactating or risk of pregnancy | Patients aged 40–50 years not on contraceptives or not being sterilised or not having had a hysterectomy |
| History of alcohol or drug abuse | Read codes for alcohol or drug abuse in the last 12 months |
| ECG abnormalities | Read code for ECG abnormalities within 12 months prior to index date |
| Long QT interval (QTc interval >450 ms for males and >470 ms for females) | Read code for long QT interval within 12 months prior to index date |
| Laboratory abnormalities (clinically relevant) | Not applied |
| Haematological laboratory abnormalities (uncontrolled) | Not applied |
| Blood chemistry abnormalities | Not applied |
| SGOT, SGPT, bilirubin or creatinine >2 × upper limit of normal | Not applied |
| Hypokalaemia | Read code for hypokalaemia within 12 months prior to index date |
| Urinalysis abnormalities | Not applied |
| X-thorax / CT abnormalities (not COPD related) | Not applied |
| Live attenuated vaccinations within 30 days | Read code for life attenuated vaccination within 30 days prior to index date |
| Irregular day/night, wake/sleep cycles, eg shift workers | Read code for shift worker ever |
| Life expectancy <1 year | QOF Read code for palliative care ever |

Abbreviations: COPD = chronic obstructive pulmonary disease; ECG = electrocardiogram; QOF = Quality and Outcomes Framework;{(HSCIC), #344} SGOT = serum glutamic oxaloacetic transaminase; SGPT = serum glutamic-pyruvic transaminase.

**Table S7** Applied definitions of exclusion criteria for specific contra-indications in the Optimum Patient Care Research Database (OPCRD)

| **RCT exclusion criterion** | **Definition in OPCRD** |
| --- | --- |
| Narrow-angle glaucoma | Read code for narrow-angle glaucoma ever |
| Symptomatic prostatic hyperplasia | Read code for prostatic hyperplasia in the last 12 months without operation to control condition |
| Bladder neck obstruction | Read code for bladder neck obstruction in the last 12 months without operation to control condition |
| History of transurethral resection of the prostate (TURP) | Read code for TURP ever |
| Renal impairment (creatinine clearance <=50 ml/min) | Read code for Stage 3B, 4 or 5 for chronic kidney disease ever |
| Hypersensitivity to LAMA | Read code for hypersensitivity to LAMA ever |
| Hypersensitivity to LABA | Read code for hypersensitivity to LABA ever |

Abbreviations: LABA = long-acting β-agonist; LAMA = long-acting muscarinic antagonist.
