# Supplementary material for: Eligibility of real-life patients with COPD for inclusion in trials of inhaled long-acting bronchodilator therapy
Source: Respir Res. 2016 Sep 23;17:120. doi: 10.1186/s12931-016-0433-5 (PMC5034631; doi:10.1186/s12931-016-0433-5)
Supplement: Additional file 6: Tables S11-S20. — Distribution of inclusion criteria in the Optimum Patient Care Research Database (OPCRD) population. Table S12. Distribution of COPD-related exclusion criteria in the population of patients with COPD in the Optimum Patient Care Research Database (OPCRD). Table S13. Distribution of concomitant pulmonary diseases in the population of patients with COPD in the Optimum Patient Care Research Database (OPCRD). Table S14. Distribution of asthma, allergic diseases and atopy in the population of patients with COPD in the Optimum Patient Care Research Database (OPCRD). Table S15. Distribution of other comorbidities in the population of patients with COPD in the Optimum Patient Care Research Database (OPCRD). Table S16. Distribution of other relevant conditions in the population of patients with COPD in the Optimum Patient Care Research Database (OPCRD). Table S17. Distribution of contra-indications in the population of patients with COPD in the Optimum Patient Care Research Database (OPCRD). Table S18. Percentage of Optimum Patient Care Research Database (OPCRD) patients with COPD and mMRC ≥2 who would be eligible for RCTs testing tiotropium (n = 17 075). Table S19. Percentage of Optimum Patient Care Research Database (OPCRD) patients with COPD and mMRC ≥2 (n = 17 075) who would be eligible for RCTs testing formoterol (F), aclidinium (A), indacaterol (I), olodaterol (O) and glycopyrronium (G). Table 20. Percentage of Optimum Patient Care Research Database (OPCRD) patients with COPD and mMRC ≥2 (n = 17 075) who would be eligible for RCTs testing indacaterol + glycopyrronium (I + G), vilanterol + umeclidinium (V + U) and tiotropium + olodaterol (T + O). (DOCX 27 kb) [file 12931_2016_433_MOESM6_ESM.docx]

# Supplementary tables

**Table S11** Distribution of inclusion criteria in the Optimum Patient Care Research Database (OPCRD) population

| **RCT inclusion criterion** | **Prevalence (number) in study population** |
| --- | --- |
| Pack years of smoking ≥ 10  Pack years of smoking ≥ 20 | 94.2% (34 758)  92.8% (34 232) |
| mMRC ≥2 | 46.3% (17 075) |
| Symptomatic^*^ | 80.2% (29 579) |
| Relatively stable COPD^†^ | 89.9% (33 167) |
| History of ≥1 exacerbation last year | 49.8% (18 373) |
| Able to perform spirometry^‡^ | 98.0% (36 142) |
| Able to record in diary^§^ | 99.9% (36 870) |

^*^MRC grade ≥2 or maintenance therapy prescribed within 6 months; ^†^No COPD exacerbations in the last 6 weeks and no changes in therapeutic regimen for COPD in the last 2 months; ^‡^No spirometry exclusion Read code after latest spirometry; ^§^No illiteracy Read code.

Abbreviations: COPD = chronic obstructive pulmonary disease; mMRC = modified Medical Research Council score.

**Table S12** Distribution of COPD-related exclusion criteria in the population of patients with COPD in the Optimum Patient Care Research Database (OPCRD)

| **RCT exclusion criterion (within period prior to index date)** | **Prevalence (number) in study population** |
| --- | --- |
| COPD exacerbation within  4 weeks  6 weeks | 5.4% (1975)  8.5% (3120) |
| Respiratory infection within  4 weeks  6 weeks | 1.6% (590)  2.8% (1031) |
| Upper respiratory infection within 6 weeks | 0.3% (127) |
| Antibiotics prescription for respiratory tract infections within 6 weeks | 2.0% (739) |
| Hospitalisation for COPD exacerbation or pneumonia within  3 months  12 months | 0.0% (18)  0.2% (83) |
| Oxygen treatment within 12 months | 0.9% (323) |
| Oral steroids use at unstable doses last 6 weeks or at stable doses of  ≥ 10 mg/day  ≥ 20 mg/day | 0.6% (214)  0.4% (153) |
| Depot corticosteroids last 12 months | 1.5% (553) |
| Active in a pulmonary rehabilitation program within  4 weeks  6 weeks  12 months | 0.1% (51)  0.2% (82)  2.9% (1083) |
| Change of maintenance treatment within 6 weeks | 1.3% (463) |
| Current use of oral sympaticomimetics | 0.0% (17) |
| Long term use of antibiotics | 3.4% (1250) |
| History of non-compliance to drugs | 15.9% (5877) |

**Table S13** Distribution of concomitant pulmonary diseases in the population of patients with COPD in the Optimum Patient Care Research Database (OPCRD)

| **RCT exclusion criterion** | **Prevalence (number) in study population** |
| --- | --- |
| Concomitant pulmonary disease ever^*^ | 10.3% (3806) |
| Clinically evident bronchiectasis ever | 5.1% (1875) |
| Lung lobectomy ever | 0.0% (1) |
| Lung transplantation ever | 0.0% (4) |
| Active tuberculosis in last 12 months | 0.1% (20) |
| Cystic fibrosis | 0.0% (8) |
| Lung cancer ever | 1.0% (387) |
| Life-threatening pulmonary obstruction ever | 0.9% (377) |
| Pulmonary embolism ever | 2.3% (861) |
| α1-antitrypsine deficiency | 0.2% (83) |

^*^Cystic fibrosis, bronchiectasis, active tuberculosis, pulmonary fibrosis, lung disease due to external agents or unspecified lung diseases

**Table S14** Distribution of asthma, allergic diseases and atopy in the population of patients with COPD in the Optimum Patient Care Research Database (OPCRD)

| **RCT exclusion criterion** | **Prevalence (number) in study population** |
| --- | --- |
| History of asthma ever | 22.8% (8415) |
| Current asthma | 3.6% (1313) |
| Blood eosinophilia >400/μl  Blood eosinophilia >600/μl | 10.0% (3690)  3.4% (1266) |
| Allergic rhinitis | 2.1% (769) |
| Allergic rhinitis treated with H1 antagonist or intra-nasal corticosteroids intermittently | 1.3% (466) |
| Atopic eczema | 9.2% (3398) |
| Anti-histamine prescribed last year | 11.7% (4304) |
| Cromolyn sodium or nedocromil sodium prescribed | 0.7% (258) |
| Atopy ever | 9.2% (3393) |

**Table S15** Distribution of other comorbidities in the population of patients with COPD in the Optimum Patient Care Research Database (OPCRD)

| **RCT exclusion criterion** | **Prevalence (number) in study population** |
| --- | --- |
| Clinically significant disease other than COPD that would put the patient at risk or a disease that would influence the results or patient’s ability to participate | 20.3% (7487) |
| Unstable cardiac conditions | 2.8% (1028) |
| Unstable angina pectoris | 0.3% (97) |
| Life-threatening (or unstable) cardiac arrhythmia in past year (not stable atrial fibrillation) | 0.0% (16) |
| Paroxysmal atrial fibrillation | 2.6% (968) |
| Paroxysmal tachycardia (>100) | 0.4% (136) |
| Myocardial infarction in past 6 months  Myocardial infarction in past 12 months | 0.4% (150)  0.9% (331) |
| Hospitalisation for heart failure (NYHA class III or IV) | 0.1% (27) |
| Left Ventricular failure (NYHA class III or IV) | 1.7% (620) |
| Beta-blockers prescribed within 3 months | 12.3% (4526) |
| Malignancy treated with resection, radiation or chemotherapy (except local skin carcinoma) within 5 years | 5.5% (2024) |
| Diabetes mellitus type 1 or uncontrolled type 2 | 0.9% (336) |
| Untreated thyrotoxicosis | 0.0% (7) |
| Hyperadrenergic state | 0.0% (1) |
| Mental illness | 15.2% (5622) |
| Tricyclic antidepressants or Monoamine oxidase inhibitors use | 11.4% (4195) |
| Uncontrolled infection due to HIV and/or active hepatitis | 0% (12) |

Abbreviations: COPD = chronic obstructive pulmonary disease; HIV = human immunodeficiency virus; NYHA = New York Heart Association.

**Table S16** Distribution of other relevant conditions in the population of patients with COPD in the Optimum Patient Care Research Database (OPCRD)

| **RCT exclusion criterion** | **Prevalence (number) in study population** |
| --- | --- |
| Pregnancy, lactating or risk of pregnancy | 1.4% (518) / 3.0% of women |
| History of alcohol or drug abuse | 5.4% (2010) |
| ECG abnormalities | 1.6% (594) |
| Long QT interval | 0.0% (2) |
| Hypokalaemia | 0.1% (48) |
| Live attenuated vaccinations within 30 days | 0.0% (2) |
| Irregular day/night, wake/sleep cycles, eg shift workers | 0.1% (21) |
| Life expectancy <1 year | 1.5% (561) |

Abbreviation: ECG = electrocardiogram.

**Table S17** Distribution of contra-indications in the population of patients with COPD in the Optimum Patient Care Research Database (OPCRD)

| **RCT exclusion criterion** | **Prevalence (number) in study population** |
| --- | --- |
| Narrow-angle glaucoma | 0.2% (57) |
| Symptomatic prostatic hyperplasia | 0% (8) |
| Bladder neck obstruction | 0.0% (1) |
| History of TURP | 0.3% (108) |
| Renal impairment (creatinine clearance ≤50 ml/min) | 18.6% (6847) |
| Hypersensitivity to LAMA | 0.0% (4) |
| Hypersensitivity to LABA | 0.0% (9) |

Abbreviations: LABA = long-acting β-agonist; LAMA = long-acting muscarinic antagonist; TURP = transurethral resection of the prostate.

**Table S18** Percentage of Optimum Patient Care Research Database (OPCRD) patients with COPD and mMRC ≥2 who would be eligible for RCTs testing tiotropium (n=17 075)

| **Step** | **Sequential selection criterion** | **NCT02172287** | **NCT00274014** | **NCT00274547** | **NCT00277264** | **NCT00144339** | **NCT00387088** | **NCT00563381** | **NCT01126437** | **NCT01455129** | **Median** |
| --- | --- | --- | --- | --- | --- | --- | --- | --- | --- | --- | --- |
| 1 | FEV_1_ | 57.9 | 58.0 | 57.9 | 66.7 | 74.7 | 57.9 | 74.7 | 74.7 | 52.9 | **58.0** |
| 2 | Other inclusion criteria | 46.6 | 25.5 | 55.3 | 29.5 | 60.3 | 55.3 | 32.0 | 61.9 | 52.9 | **52.9** |
| 3 | COPD-related exclusion criteria | 45.6 | 24.4 | 51.0 | 27.2 | 58.8 | 54.0 | 31.6 | 60.1 | 47.0 | **47.0** |
| 4 | Concomitant pulmonary disease | 42.5 | 24.4 | 51.0 | 25.0 | 51.2 | 50.3 | 31.2 | 52.2 | 40.6 | **42.5** |
| 5 | Asthma, allergic diseases and atopy | 27.9 | 17.5 | 40.0 | 18.2 | 40.4 | 36.1 | 24.1 | 41.2 | 29.8 | **29.8** |
| 6 | Comorbidities | 19.1 | 13.3 | 37.1 | 11.5 | 30.0 | 33.5 | 18.2 | 30.5 | 22.4 | **22.4** |
| 7 | Other relevant conditions | 17.8 | 13.3 | 37.1 | 11.4 | 28.0 | 33.5 | 17.2 | 28.5 | 20.8 | **20.8** |
| **8** | **Final eligible proportion (%)** | **17.8** | **13.3** | **29.2** | **11.4** | **22.2** | **33.5** | **13.6** | **22.6** | **16.7** | **17.8** |

Abbreviations: COPD = chronic obstructive pulmonary disease; FEV_1_ = forced expiratory volume in 1 second.

**Table S19** Percentage of Optimum Patient Care Research Database (OPCRD) patients with COPD and mMRC ≥2 (n=17 075) who would be eligible for RCTs testing formoterol (F), aclidinium (A), indacaterol (I), olodaterol (O) and glycopyrronium (G)

| **Step** | **Sequential selection criterion** | **NCT00134979 (F)** | **NCT00363896 (A)** | **NCT01001494 (A)** | **NCT01044459 (A)** | **NCT00463567 (I)** | **NCT00567996 (I)** | **NCT00792805 (I)** | **NCT00845728 (I)** | **NCT00782210 (O)** | **NCT00793624 (O)** | **NCT00929110 (G)** | **NCT01005901 (G)** | **NCT01566604 (G)** | **Median** |
| --- | --- | --- | --- | --- | --- | --- | --- | --- | --- | --- | --- | --- | --- | --- | --- |
| 1 | FEV_1_ | 73.1 | 86.1 | 79.1 | 79.1 | 79.1 | 79.1 | 79.1 | 30.3 | 86.1 | 86.1 | 79.1 | 79.1 | 79.1 | **79.1** |
| 2 | Other inclusion criteria | 60.6 | 71.4 | 65.8 | 65.8 | 74.3 | 74.3 | 75.1 | 17.8 | 81.9 | 81.9 | 75.0 | 75.1 | 73.2 | **74.3** |
| 3 | COPD-related exclusion criteria | 60.3 | 69.7 | 65.1 | 64.3 | 58.1 | 71.0 | 65.5 | 14.1 | 80.6 | 79.5 | 71.4 | 72.5 | 63.8 | **65.5** |
| 4 | Concomitant pulmonary disease | 52.8 | 62.1 | 58.1 | 57.4 | 52.1 | 63.4 | 57.7 | 12.1 | 79.6 | 74.4 | 63.6 | 63.5 | 57.0 | **58.1** |
| 5 | Asthma, allergic diseases and atopy | 41.5 | 44.4 | 45.5 | 45.0 | 41.1 | 44.9 | 45.2 | 9.5 | 77.3 | 69.7 | 48.1 | 49.5 | 43.0 | **45.0** |
| 6 | Comorbidities | 31.1 | 42.9 | 43.8 | 36.4 | 29.9 | 34.8 | 42.3 | 9.0 | 56.1 | 64.4 | 34.8 | 47.6 | 31.4 | **36.4** |
| 7 | Other relevant conditions | 30.9 | 41.7 | 43.8 | 36.4 | 29.7 | 33.9 | 42.3 | 9.0 | 56.1 | 59.7 | 34.6 | 47.6 | 30.9 | **36.4** |
| 8 | **Final eligible proportion (%)** | **30.9** | **41.6** | **43.7** | **36.3** | **23.3** | **33.9** | **42.3** | **9.0** | **56.1** | **59.7** | **27.2** | **37.0** | **24.2** | **36.3** |

Abbreviations: Abbreviations: COPD = chronic obstructive pulmonary disease; FEV_1_ = forced expiratory volume in 1 second.

**Table 20** Percentage of Optimum Patient Care Research Database (OPCRD) patients with COPD and mMRC ≥2 (n=17 075) who would be eligible for RCTs testing indacaterol+glycopyrronium (I+G), vilanterol+umeclidinium (V+U) and tiotropium+olodaterol (T+O)

| **Step** | **Sequential selection criterion** | **NCT01120691 (I+G)** | **NCT01202188 (I+G)** | **NCT01315249 (I+G)** | **NCT01709903 (I+G)** | **NCT01782326 (I+G)** | **NCT01313650 (V+U)** | **NCT01316913 (V+U)** | **NCT01777334 (V+U)** | **NCT01431287 (T+O)** | **Median** |
| --- | --- | --- | --- | --- | --- | --- | --- | --- | --- | --- | --- |
| 1 | FEV_1_ | 37.3 | 79.1 | 66.2 | 79.1 | 53.7 | 74.7 | 74.7 | 74.7 | 86.1 | **74.7** |
| 2 | Other inclusion criteria | 22.2 | 75.0 | 61.2 | 73.1 | 29.8 | 71.2 | 71.2 | 71.2 | 81.9 | **71.2** |
| 3 | COPD-related exclusion criteria | 13.7 | 53.9 | 22.6 | 46.4 | 23.2 | 61.5 | 61.0 | 68.7 | 79.5 | **53.9** |
| 4 | Concomitant pulmonary disease | 11.9 | 47.5 | 20.3 | 41.9 | 20.2 | 54.7 | 54.3 | 60.1 | 74.4 | **47.5** |
| 5 | Asthma, allergic diseases and atopy | 7.8 | 31.3 | 14.0 | 32.1 | 15.1 | 43.1 | 52.8 | 58.3 | 72.2 | **32.1** |
| 6 | Comorbidities | 5.7 | 23.6 | 10.9 | 24.2 | 10.8 | 31.9 | 39.3 | 43.4 | 52.5 | **24.2** |
| 7 | Other relevant conditions | 5.6 | 22.8 | 10.8 | 23.9 | 10.4 | 29.5 | 36.2 | 40.0 | 49.0 | **23.9** |
| 8 | **Final eligible proportion (%)** | **4.7** | **17.9** | **8.3** | **18.6** | **8.4** | **74.7** | **28.6** | **31.7** | **49.0** | **18.6** |

Abbreviations: COPD = chronic obstructive pulmonary disease; FEV_1_ = forced expiratory volume in 1 second.
